# Supplementary material for: Ancient DNA from Protohistoric Period Cambodia indicates that South Asians admixed with local populations as early as 1st–3rd centuries CE
Source: Sci Rep. 2022 Dec 29;12:22507. doi: 10.1038/s41598-022-26799-3 (PMC9800559; doi:10.1038/s41598-022-26799-3)
Supplement: Supplementary file 1 — Supplementary Information 1. [file 41598_2022_26799_MOESM1_ESM.pdf]

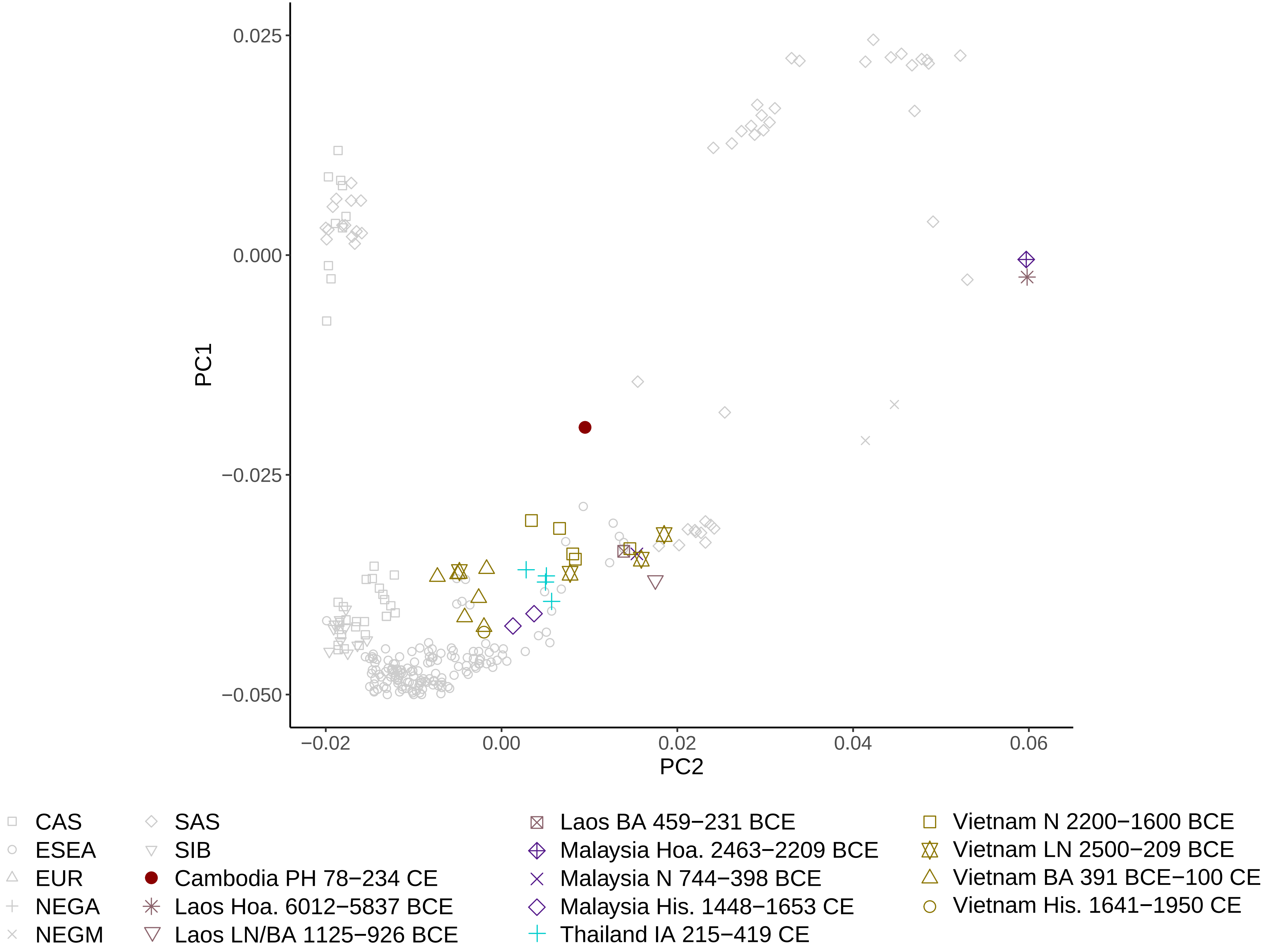

0.025
0.000
−0.025
−0.050
PC1
−0.02
0.00
0.02
0.04
0.06
PC2
CAS
ESEA
EUR
NEGA
NEGM
SAS
SIB
Cambodia PH 78−234 CE
Laos Hoa. 6012−5837 BCE
Laos LN/BA 1125−926 BCE
Laos BA 459−231 BCE
Malaysia Hoa. 2463−2209 BCE
Malaysia N 744−398 BCE
Malaysia His. 1448−1653 CE
Thailand IA 215−419 CE
Vietnam N 2200−1600 BCE
Vietnam LN 2500−209 BCE
Vietnam BA 391 BCE−100 CE
Vietnam His. 1641−1950 CE
