# Supplementary figures and images for: Ancient DNA from Protohistoric Period Cambodia indicates that South Asians admixed with local populations as early as 1st–3rd centuries CE
Source: Sci Rep. 2022 Dec 29;12:22507. doi: 10.1038/s41598-022-26799-3 (PMC9800559; doi:10.1038/s41598-022-26799-3)

$f_4$ (Orcadian, Japanese; an ancient MSEA individual, all other ancient MSEA)

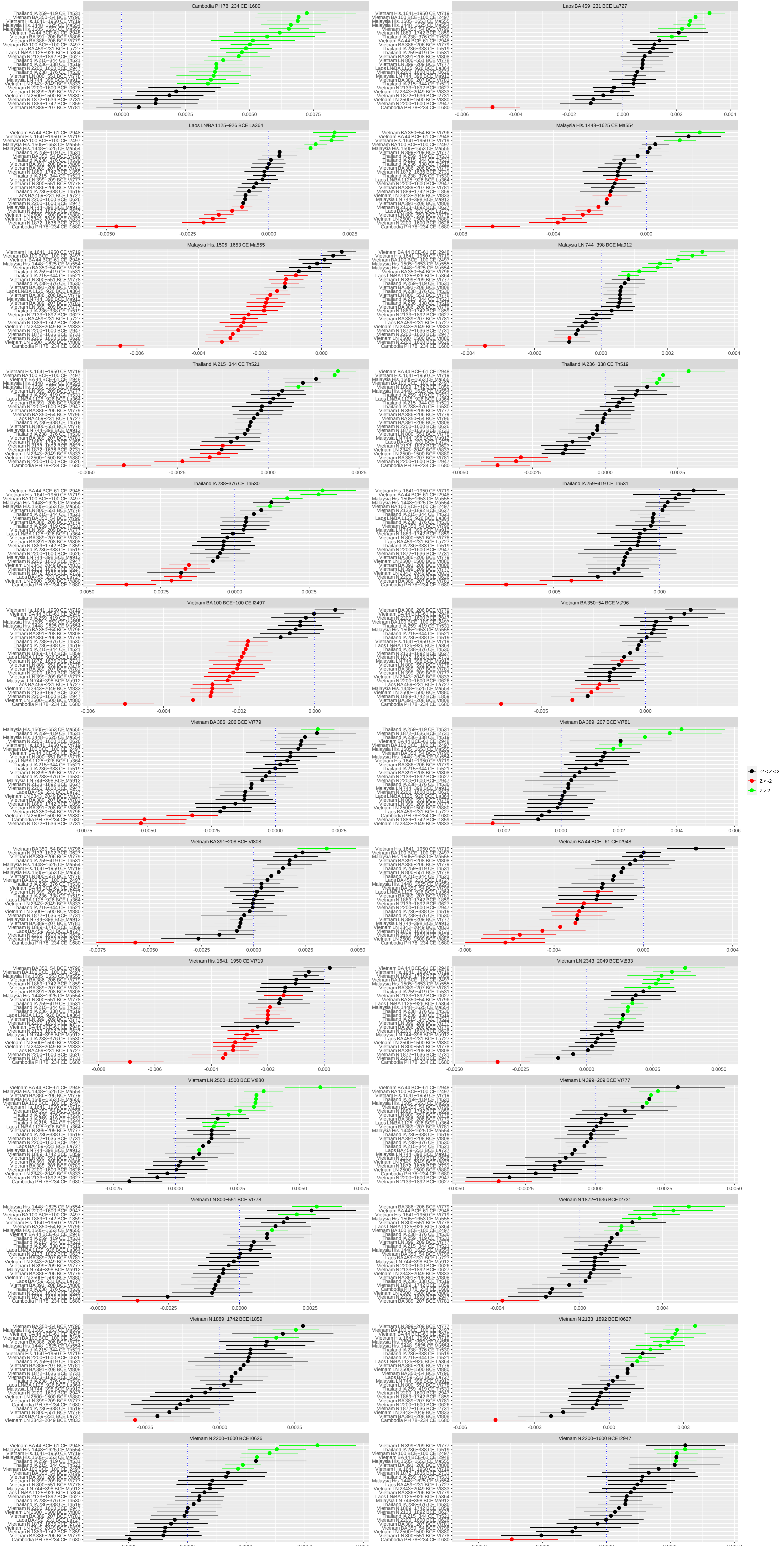

Supplement: Supplementary file 2 — Supplementary Information 2. [file 41598_2022_26799_MOESM2_ESM.pdf]

**$f_4$ (Vellalar, Japanese; an ancient MSEA individual, all other ancient MSEA)**

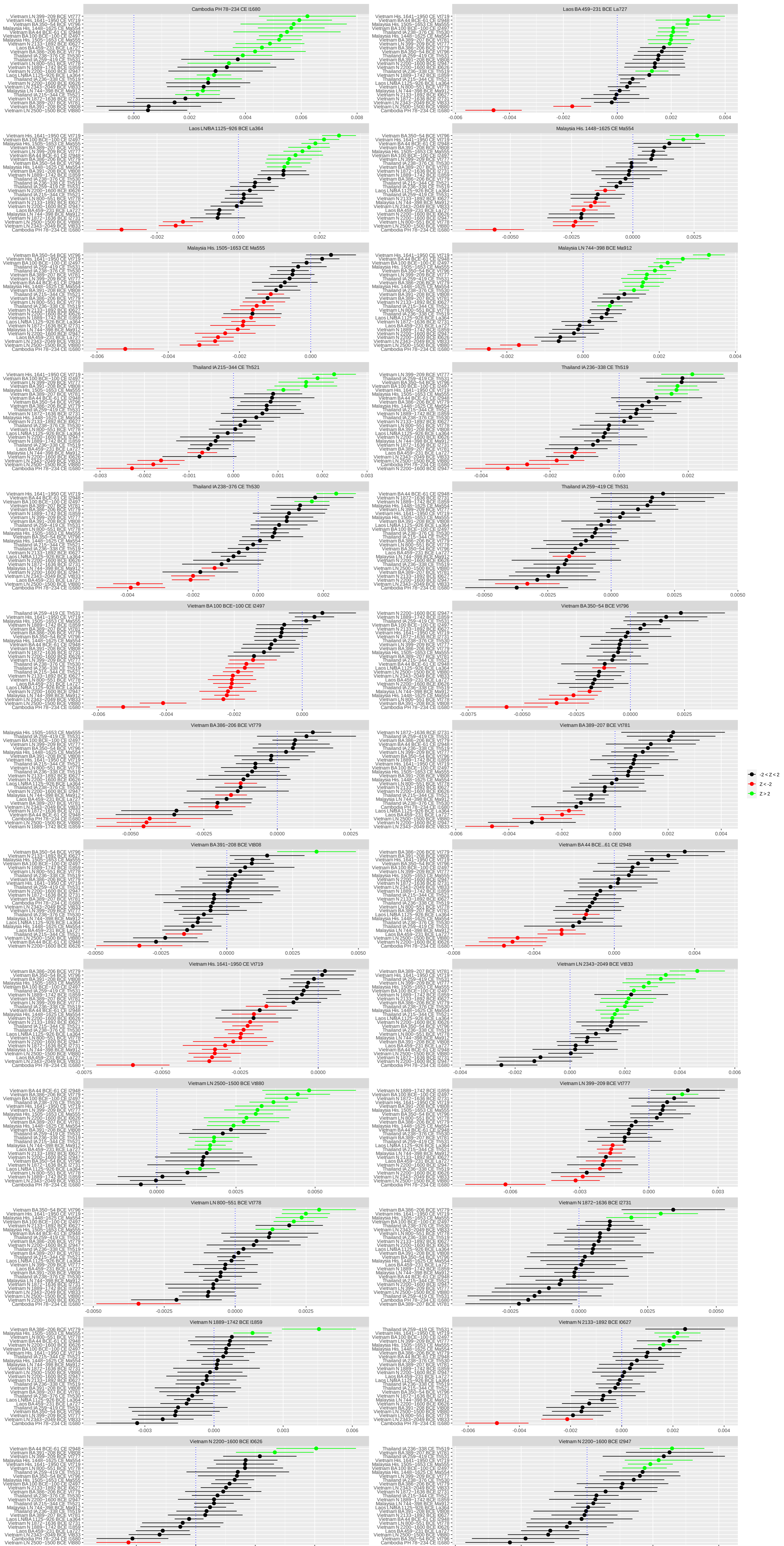

Supplement: Supplementary file 3 — Supplementary Information 3. [file 41598_2022_26799_MOESM3_ESM.pdf]

$f_4$ (an ESEA group, all other ESEA; Cambodia PH, present-day Cambodians)

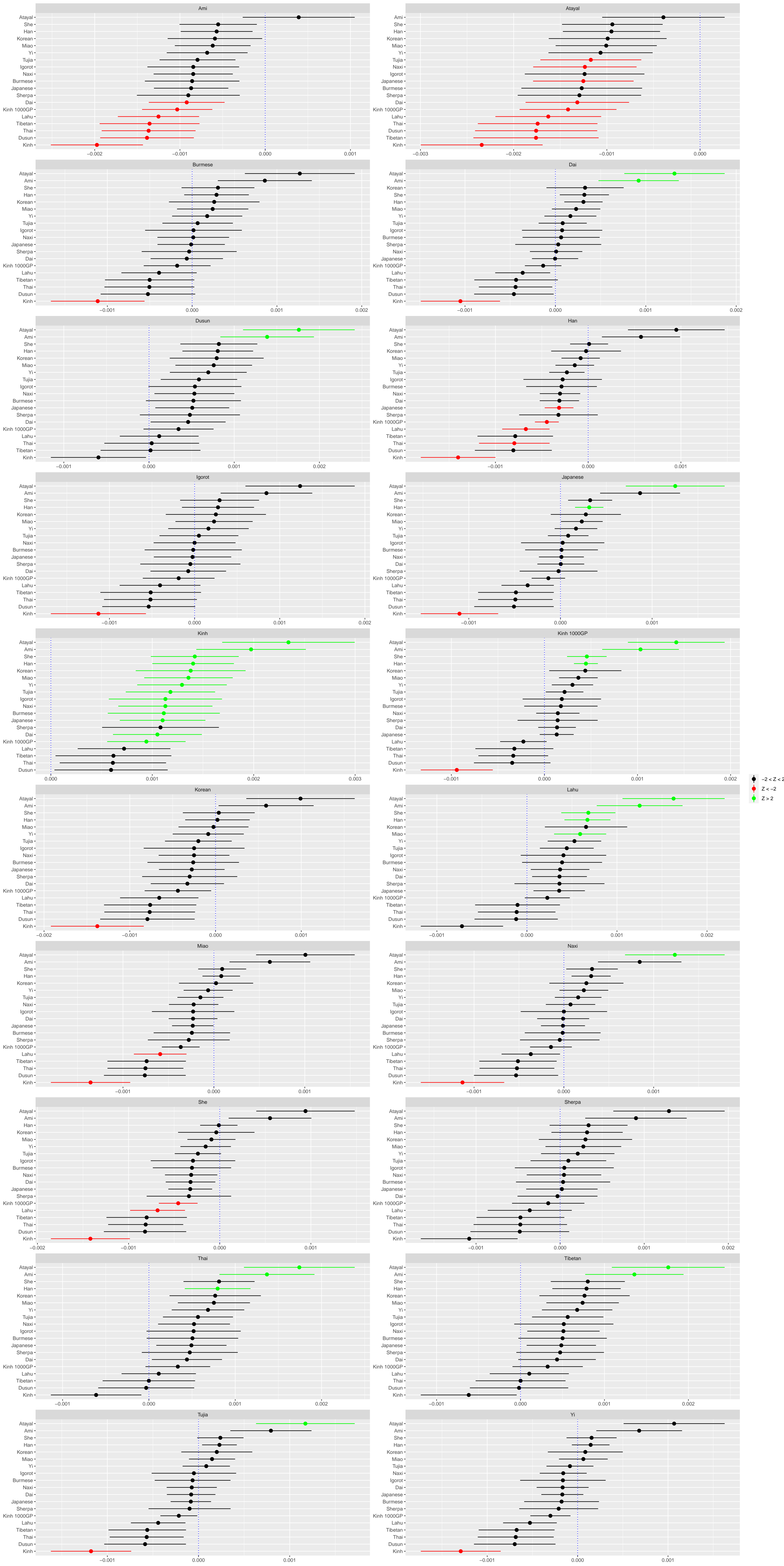

Supplement: Supplementary file 4 — Supplementary Information 4. [file 41598_2022_26799_MOESM4_ESM.pdf]

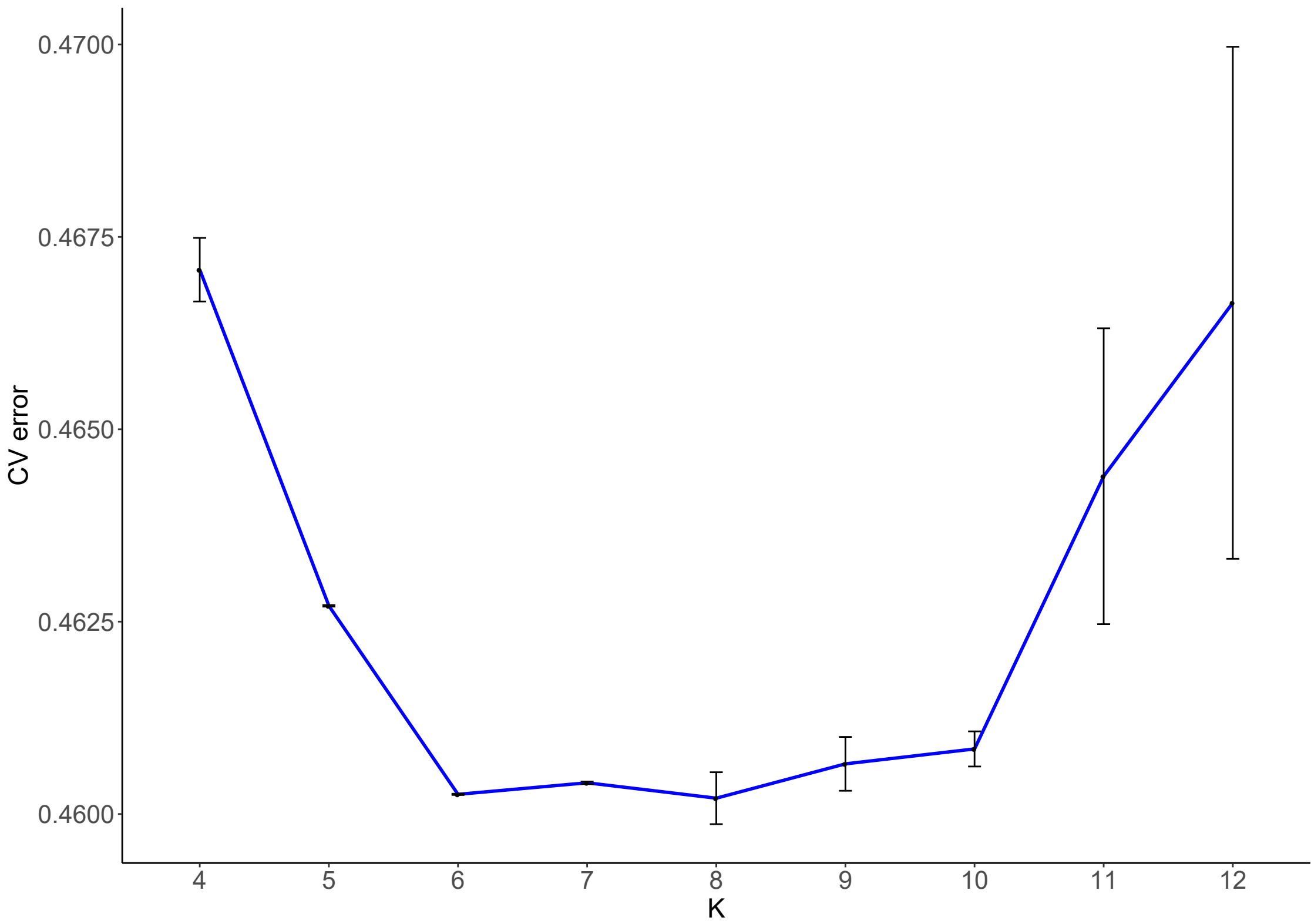

Supplement: Supplementary file 6 — Supplementary Information 6. [file 41598_2022_26799_MOESM6_ESM.pdf]
