# Supplementary material for: Ancient DNA from Protohistoric Period Cambodia indicates that South Asians admixed with local populations as early as 1st–3rd centuries CE
Source: Sci Rep. 2022 Dec 29;12:22507. doi: 10.1038/s41598-022-26799-3 (PMC9800559; doi:10.1038/s41598-022-26799-3)
Supplement: Supplementary file 5 — Supplementary Information 5. [file 41598_2022_26799_MOESM5_ESM.pdf]

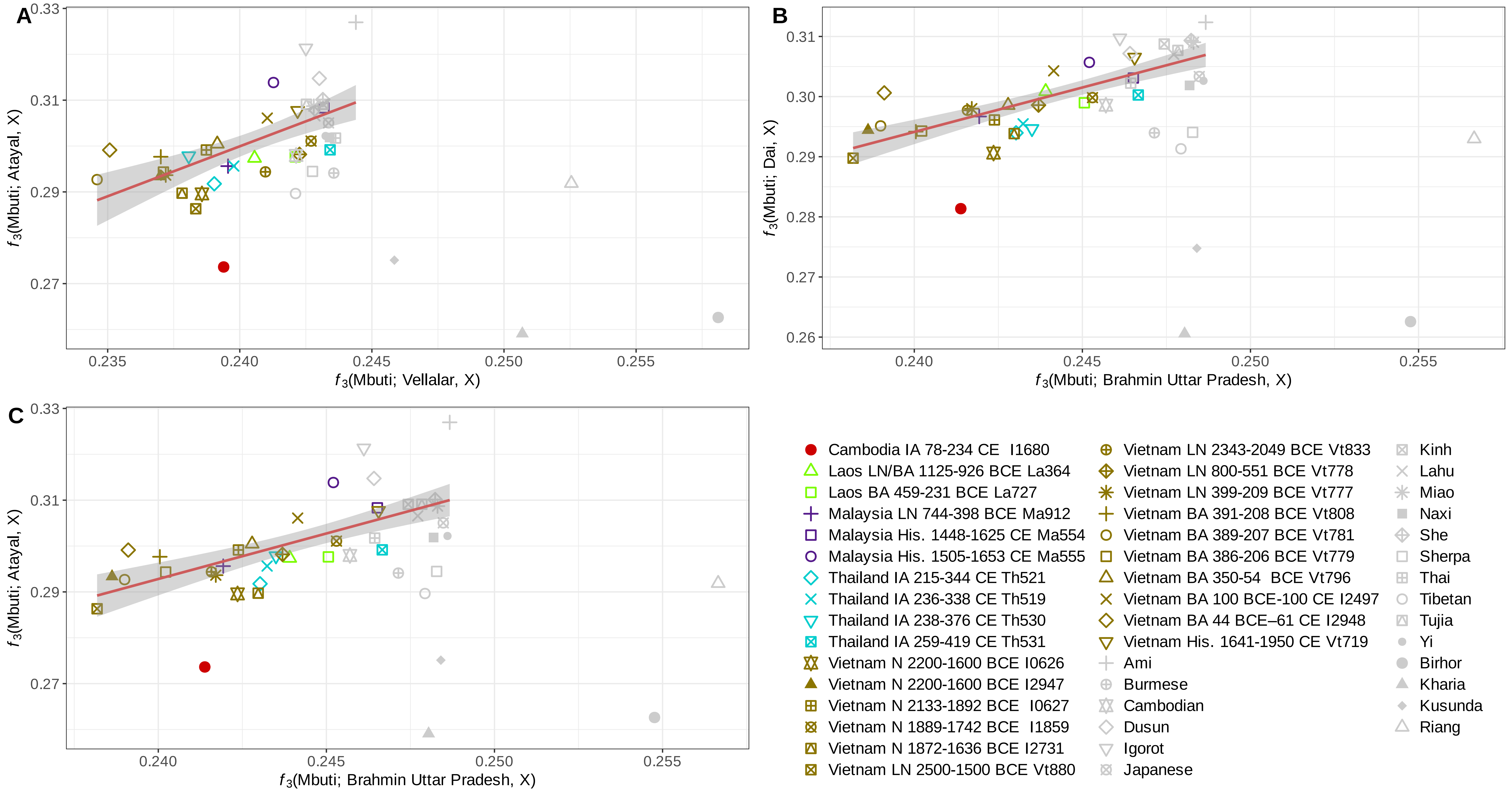
A
B
C
0.33
0.31
0.29
0.27
0.26
0.28
0.30
0.235
0.240
0.245
0.250
0.255
f_3(Mbuti; Atayal, X)
f_3(Mbuti; Vellalar, X)
f_3(Mbuti; Dai, X)
f_3(Mbuti; Brahmin Uttar Pradesh, X)
Cambodia IA 78-234 CE I1680
Laos LN/BA 1125-926 BCE La364
Laos BA 459-231 BCE La727
Malaysia LN 744-398 BCE Ma912
Malaysia His. 1448-1625 CE Ma554
Malaysia His. 1505-1653 CE Ma555
Thailand IA 215-344 CE Th521
Thailand IA 236-338 CE Th519
Thailand IA 238-376 CE Th530
Thailand IA 259-419 CE Th531
Vietnam N 2200-1600 BCE I0626
Vietnam N 2200-1600 BCE I2947
Vietnam N 2133-1892 BCE I0627
Vietnam N 1889-1742 BCE I1859
Vietnam N 1872-1636 BCE I2731
Vietnam LN 2500-1500 BCE Vt880
Vietnam LN 2343-2049 BCE Vt833
Vietnam LN 800-551 BCE Vt778
Vietnam LN 399-209 BCE Vt777
Vietnam BA 391-208 BCE Vt808
Vietnam BA 389-207 BCE Vt781
Vietnam BA 386-206 BCE Vt779
Vietnam BA 350-54 BCE Vt796
Vietnam BA 100 BCE-100 CE I2497
Vietnam BA 44 BCE–61 CE I2948
Vietnam His. 1641-1950 CE Vt719
Ami
Burmese
Cambodian
Dusun
Igorot
Japanese
Kinh
Lahu
Miao
Naxi
She
Sherpa
Thai
Tibetan
Tujia
Yi
Birhor
Kharia
Kusunda
Riang
